# Leisure experience and mobile phone addiction: Evidence from Chinese adolescents

**DOI:** 10.1016/j.heliyon.2024.e24834

**Published:** 2024-01-17

**Authors:** Ximei Xia, Shuhui Qin, Shiyin Zhang

**Affiliations:** Department of Psychology, Qingdao University, Qingdao, 266071, China

## Abstract

**Background:**

During the Covid-19 pandemic, online learning became the mainstream because of many restrictions on interpersonal relationships. Children spent more and more time using mobile phones, which also aroused public concern.

In past research on the prevention of problematic mobile phone use, it was easy to neglect meaningful part of leisure. Hence, based on Davis's cognitive-behavioral model, this study was designed to verify how leisure experience influences mobile phone addiction through maladaptive cognition, which received little attention before.

**Methods:**

By convenient sampling method, it involved a sample of 1007 middle school students recruited from Northern China. We used adolescent leisure experience questionnaire, maladaptive cognition scale and mobile phone addiction scale to measure adolescents' leisure experience, maladaptive cognition and mobile phone addiction respectively.

**Results:**

The findings revealed that leisure experience was negatively correlated with maladaptive cognition (r = −0.21, p < 0.01) and mobile phone addiction (r = −0.20, p < 0.01) respectively. Maladaptive cognition was positively correlated with mobile phone addiction (r = 0.51, p < 0.01). Given gender, age and family economic conditions, the negative predictive effect of leisure experience on mobile phone addiction was significant (β = −0.18，p < 0.001). Besides, the process by which leisure experience predicted mobile phone addiction through maladaptive cognition was significant, indirect effect = −0.10, SE = 0.02, 95 % CI = [−0.13, −0.07].

**Conclusions:**

Adolescents’ great leisure experience has a positive impact on mobile phone addiction, which can be achieved by reducing maladaptive cognition. Therefore，it is significant to improve their leisure experience and guide them to perceive their irrational beliefs in leisure and rethink offline leisure and real life from more positive views.

## 1 Introduction

Technological upheaval has a profound influence on people's lives. In modern society, as a momentous means of information transmission, mobile phones have gained a strong position as a sign of communication technology [1]. During the Covid-19 pandemic, online learning became the mainstream. Children easily found the learning materials they want and used social media or played online games to relax through mobile phones.

However, the mobile phone is also a double-edged sword. Despite its great convenience, the problem caused by the mobile phone has raised concerns among the public and researchers, such as mobile phone addiction among adolescents [2].Many parents worry that their offspring are addicted to online games or short-form videos, which affect normal study [3].

Mobile phone addiction not only harms physical health, but also negatively affects individual psychological and social functions. For example, researchers have found that mobile phone addiction is related to poor sleep quality, anxiety, and depression [4,5]. Furthermore, problematic smartphone use may harm academic performance [6].

In summary, we realize that it is essential to determine how some factors contribute to mobile phone addiction. Therefore, our study aimed to explore the relationship between leisure experience and mobile phone addiction, as well as the mediating role of maladaptive cognition.

### 1.1 Leisure benefits

Past researches have shown that both external contributors (e.g., childhood experiences, interpersonal aspects) and individual contributors (e.g., personality, attachment, self-control) arouse mobile phone addiction [2,[7], [8], [9], [10]]. Nevertheless, there are relatively few studies covering the meaningful part of leisure in the development and prevention of mobile phone addiction. Leisure, as an important way of life to enrich people's spiritual world, is gradually favored by the public and researchers.

Studies have demonstrated positive roles of leisure in influencing health status. Apart from maintaining and improving physical health, it is well-established that leisure activities are also beneficial to mental health. Specifically, physical activities reduce the risk factors of cardiovascular disease in adolescents, such as triglyceride [11]; an empirical study from the UK found that, after considering factors such as weight, higher moderate-to-vigorous physical activity in adolescents was related to decreased hyperactivity score, peer problems and depressive-symptoms [12]. Besides, Mock and Smale confirmed that social leisure activities (e.g., watching sports events on the spot), cultural leisure activities (e.g., visiting some museums) and exercise are good for life satisfaction and wellbeing [13].

Such active leisure activities have been proven to have a positive impact on mobile phone addiction. A cross-sectional study showed that physical exercise has a negative predictive effect on college students' mobile phone addiction. However, what factors in leisure make a difference to adolescents, and what quality of leisure produces better results? These problems need further study.

Therefore, we consider that it is worth discussing how leisure and some perceptions of it affect mobile phone addiction and it is also an important research issue in this paper.

### 1.2 Leisure experience and mobile phone addiction

Adolescents gain positive experience through using their phones, which is very important for them. The experiences mainly include emotion improvement and need satisfaction. They relieve or get rid of negative emotions such as depression by immersing themselves in content and activities that bring timely satisfaction and happiness. They spend more time on mobile phones to disperse their negative feelings [14]. In addition, in the virtual world, adolescents freely choose what they are interested in, post photos or videos on social media to gain recognition from other users and interact with them online. Obviously, these are only part of it. The behaviors increase adolescents' sense of freedom, accomplishment and belonging, and meet their psychological needs [15].

However, many applications in mobile phones provide highly immersive services, aiming at attracting the attention of users with certain characteristics as much as possible and prolonging their use time [16]. Adolescents are one of their targets. Their self-control ability is not yet mature, and they pay little attention to the value of long-term goals and the future consequences of their current behavior, so they may often find it difficult to monitor their behavior and resist mobile phones that bring direct and quick pleasure to them [17,18]. When the online benefits become the main or only source of adolescents' positive experiences, the use of mobile phones may have a negative impact on mental health, leading to the development of mobile phone addiction.

Mobile phone addiction, also called problematic mobile phone use, means an addictive behavior that causes adverse consequences to individual physiology and psychology due to inappropriate and overuse of mobile phones [19]. Like other Internet-related addiction behaviors, mobile phone addiction also involves some symptoms similar to addiction, such as losing control of mobile phone use, psychological withdrawal when unable to use mobile phone, craving for mobile phone use [20].

As a significant part of a healthy life, active leisure activities may be a potentially effective way to prevent and reduce behavior addiction. For example, Li et al. found that adolescents' physical exercise was negatively correlated with problematic mobile phone use [21]. We are curious about how the psychological state behind leisure activities affects the positive result.

In recent years, researchers are increasingly interested in individual psychological experience in leisure activities, such as pleasure, satisfaction and leisure motivation [22]. Different from leisure participation, leisure experience reflects various emotions and perceptions commonly associated with individual leisure [23]. Individual perceptions of freedom, competence, and intrinsic motivation in leisure participation are essential in many leisure experiences [24,25].

In accordance with the compensatory satisfaction theory, individuals with low reality satisfaction but high Internet satisfaction of psychological needs were most likely to develop Internet addiction [26]. We infer from it that adolescents who discover the benefits of the real world in meeting their needs are less likely to tend to look for satisfaction online, thus reducing the use of mobile phones. When leisure participants have clear leisure goals, regard the ongoing leisure activities as challenging, and devote themselves wholeheartedly, they constantly surpass themselves, and then gain the sense of pleasure, control, and accomplishment, which means they gain better leisure experience [27]. This shows that positive leisure experiences partially replace the positive effects of mobile phone use and increase the internal resources of individuals. For adolescents, positive leisure experience is extremely important [28]. It reduces adolescents' tendency to seek positive emotions and needs to be satisfied online, thus preventing them from mobile phone addiction. On the contrary, many researchers confirmed that poor leisure experience is a significant incentive for adolescents to use the mobile phone more frequently [29,30].

In conclusion, we propose that the better adolescents' leisure experience, the lower their mobile phone addiction.

### 1.3 The mediation of maladaptive cognition

Davis proposed a cognitive-behavioral model explained that Pathological Internet Use (PIU) is the result of interaction between distal contributory causes and proximal contributory causes [31]. According to the model, distal contributory causes include psychopathology (such as, depression, social anxiety, and substance dependence, etc.) and situational cues; proximal contributory causes refer to maladaptive cognition of Internet. This theoretical conception is also supported by many empirical studies [32,33].

The distal contributory causes act through the proximal contributory causes. Specifically, in our study, distal contributory cause (leisure experience) may affect mobile phone addiction through proximal contributory cause (maladaptive cognition): unsatisfactory leisure experience, often referred to a situational variable, is positively associated with the degree of mobile phone addiction [29]. As a negative cognitive factor, maladaptive cognition reflects negative views on the real world and real self, as well as the positive views on the virtual world and virtual self [31]. Previous studies have revealed that maladaptive cognition is negatively correlated with Internet-related behavioral addiction [33].

Although all the above analyses have shown that there is a relationship between leisure experience, maladaptive cognition and mobile phone addiction, the mechanism underlying it is still obscure. Hence, the aim is to investigate the possible mechanisms behind it.

First of all, as a distal situational cue, leisure experience impacts maladaptive cognition. Poor leisure experience may allow adolescents to form negative views of real society and themselves. In the real life, adolescents with poor leisure experience may feel that they are unfree, miserable and incapable in leisure. In other words, they perceive a loss of control over the situation, which leads to the formation of negative realistic self-perception, such as lower self-esteem and self-worth [34]. They are highly likely to seek help from mobile phones and the Internet for the sake of escaping from the bad reality society and changing such negative perception. The anonymity and editability of the Internet enable them to shake off the negative experiences and obtain positive feedback in the virtual world, thus forming a positive perception of the virtual society and themselves [35].

Moreover, as a proximal contributory cause, perhaps the presence of maladaptive cognition is foremost element of the cognitive-behavioral model and has a powerful impact on mobile phone addiction [31]. Maladaptive cognition distorts adolescents' views on the real and virtual world, and makes their lives focus on the Internet and mobile phone. Their sense of control and self-worth is found in the virtual world. Due to the availability and convenience of mobile phones, adolescents may rely too much on mobile phones to gratify the needs, resulting in an increase in the danger of mobile phone addiction. Particularly, when they initially try a new Internet feature, adolescents are reinforced by the positive response and are then conditioned to use their mobile phones more often to earn the same response as the one related to the initial event. With the passage of time, the use of mobile phones, the positive results after use, and the expectation of mobile phone use are getting closer and closer. Many empirical studies have shown that maladaptive cognition is closely related to mobile phone addiction [32,33].

In contrast, individuals with higher leisure experience are satisfied with psychological needs in leisure, and they have a more positive view of the real world and themselves. It means that there is no need to get compensation from the Internet, thus preventing and reducing excessive mobile phones use.

### 1.4 The present study

Overall, the fundamental purpose of our study is to develop previous research regarding adolescents’ leisure experience and mobile phone addiction according to the compensatory satisfaction theory and the cognitive-behavioral model mentioned above. The further research that we examined whether maladaptive cognition is the mediating variable in both. Therefore, we developed a mediation model to test the two hypotheses presented below.

Hypothesis 1 Leisure experience is negatively associated with mobile phone addiction.

Hypothesis 2 Leisure experience influences mobile phone addiction through maladaptive cognition. Leisure experience is negatively correlated with maladaptive cognition while maladaptive cognition is positively correlated with mobile phone addiction.

**Fig. 1:**
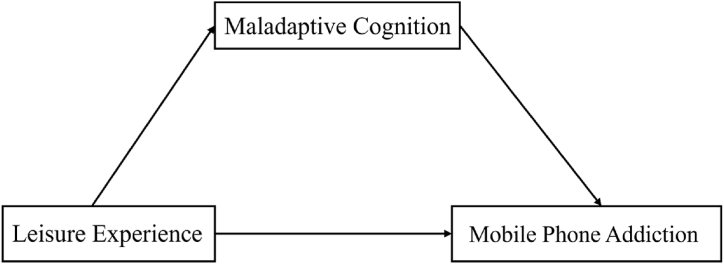
The theoretical mediation model.

The theoretical model is demonstrated in Fig. 1.

## 2 Methods

### 2.1 Procedure

Our study began in September 2022. There was an hour-long session once a week to discuss our research progress. Considering the problem of adolescents' mobile phone use was a hot topic of concern to schools, families and society, and leisure was our team's research orientation, so we referred to relevant literature and combine the two to determine the specific research problems.

According to previous studies, we used questionnaire survey. The full questionnaire includes the following demographic characteristics: (1) Gender, (2) Age, (3) Grade, (4) Family economic conditions. In addition, we used three scales, adolescent leisure experience questionnaire, maladaptive cognition scale，mobile phone addiction scale, to measure adolescents' leisure experience, maladaptive cognition and mobile phone addiction respectively.

It aimed to guarantee authenticity and validity of the questionnaire, we conducted prior training for the research team members and teachers. Before the test, the experimenters made sure the subjects fully understand the questionnaire instructions and confidentiality principle, and then arranged them to accomplish the questionnaire.

### 2.2 Participants

By convenient sampling method, 1007 students from grade 7 to 9 recruited from a junior high school in Northern China were tested. Considering that the subjects are young and easy to lose concentration, the paper questionnaire will be better than the electronic questionnaire. After testing in the field, we returned all questionnaires directly. The following are our exclusion criteria for selecting participants: (1) Eliminated abnormal questionnaires, such as regular responses. (2) Excluded participants with missing more than 3 questions. At last, there were 979 valid questionnaires (97.22 %).

The participants involved students between the ages of 12 and 17 (13.49 ± 1.01). 488 (49.85 %) were male and 491 (50.15 %) were female. There were 321 (32.79 %), 340 (34.73 %) and 318 (32.48 %) participants in grades 7 through 9. In addition, there were 134 (13.69 %) students with excellent family economic conditions, 613 (62.61 %) students with good family economic conditions, 220 (22.47 %) students with average family economic conditions and 12 (1.23 %) students with poor family economic conditions (Table 1).

**Table 1:** Participants’ demographic characteristics.

VARIABLES	FREQUENCY	PERCENTAGE
**GENDER**		
Male	488	49.8
Female	491	50.2
**AGE**		
12	182	18.6
13	316	32.3
14	306	31.3
15	168	17.2
16	6	0.6
17	1	0.1
**GRADE**		
Grade 7	321	32.8
Grade 8	340	34.7
Grade 9	318	32.5
**FAMILY ECONOMIC CONDITIONS**		
Excellent	134	13.7
Good	613	62.6
Average	220	22.5
Poor	12	1.2

### 2.3 Measures

#### 2.3.1 Leisure experience

The adolescent leisure experience questionnaire compiled by Yu was used to evaluate the level of leisure experience of the subjects, and it has good reliability and validity [36]. It included 21 items with four dimensions: (1) Perceived freedom (4 items, “I am free to spend my leisure time”); (2) Perceived competence (4 items, “I can get a sense of achievement in leisure activities.”); (3) Perceived intrinsic motivation (8 items, “I enjoy the process of doing leisure activities.”); (4) Perceived extrinsic motivation (5 items, “I do leisure activities mainly to win prizes or get everyone's affirmation.”). Based on the definition of leisure experience in this study, the first three factors of the questionnaire were used to access the leisure experience of the participants. The higher the score on all three dimensions, the better the leisure experience. The five-point scoring method is adopted, from “complete inconformity” to “complete conformity”, which is recorded as 1 to 5 points. Cronbach's alpha value of the questionnaire in our study was 0.93 and Cronbach's alpha values of the three dimensions were 0.86, 0.87 and 0.91, respectively, indicating good reliability. Additionally, we performed confirmatory factor analysis on the questionnaire and obtain the following fitting indices: χ^2^/df = 6.26, RMSEA = 0.073, CFI = 0.95, TLI = 0.94, NFI = 0.94, RFI = 0.93, and IFI = 0.95, which show construct validity is acceptable.

#### 2.3.2 Maladaptive cognition

This study adopted the maladaptive cognition scale in Chinese with good reliability and validity [31]. The scale, a 5-point Likert scale from 1 (never) to 5 (always), consists of 7 items (e.g. “The Internet is my only friend.”). The higher the score, the higher level of maladaptive cognition. In our study, the Cronbach's alpha was α = 0.96.

#### 2.3.3 Mobile phone addiction

The mobile phone addiction scale measured the degree of mobile phone addiction [37]. It contains three dimensions with 11 items in total (e.g., “While not using the mobile phone, I still think about using the mobile phone and have visions about using the mobile phone.”). It is a Likert scale, with 1–6 being from “incompletely agreed” to “completely agreed”. The higher the score, the higher degree of mobile phone addiction. In our research, the Cronbach's alpha of the total scale was 0.92.

### 2.4 Statistical analysis

First, SPSS 23.0 was used in this study to establish a database and conducted statistical analysis. The Harman's one-way test was used first on the raw data and prevent possible common method bias due to the self-reported questionnaires of the subjects [38]. Next, Cronbach's alpha coefficients and some factors from confirmatory factor analysis were used to evaluate the reliability and validity of all questionnaires through SPSS 23.0 and Amos 22.0. Then, we conducted descriptive statistics, including standard deviation and correlation, etc. Eventually, the mediation model of maladaptive cognition was examined by SPSS macro PROCESS which was developed by Hayes [39].

## 3 Results

### 3.1 The common method bias examination

The Harman's one-way method was conducted, and there are 7 factors with eigenvalues greater than 1. They contribute 74.42 % to the total variance. The first of these factors accounted for 31.42 %, lower than the critical standard 40 %. Hence, there was no serious common method bias in the study.

### 3.2 Descriptive statistics

The means, standard deviations and correlations of all variables are displayed in Table 2. As it showed, leisure experience was negatively correlated with maladaptive cognition (r = −0.21, p < 0.01) and mobile phone addiction (r = −0.20, p < 0.01) respectively; maladaptive cognition was positively correlated with mobile phone addiction (r = 0.51, p < 0.01). Given the potential impact of the variables on the theoretical model, gender, age and family economic conditions were included as covariates.

**Table 2:** Descriptive statistics and correlation coefficients (N = 979). *p < 0.05, **p < 0.01. FEC = Family Economic Conditions; PF = Perceived Freedom; PC = Perceived Competence; PIM = Perceived Intrinsic Motivation; LE = Leisure Experience; MC = Maladaptive Cognition; MPA = Mobile Phone Addiction, the following were the same.

Variables	Mean	SD	1	2	3	4	5	6	7	8	9
1.Gender	1.50	0.50	1								
2.Age	13.49	1.01	−0.01	1							
3.FEC	2.11	0.63	−0.02	0.12**	1						
4.PF	4.16	0.89	−0.02	−0.04	−0.17**	1					
5.PC	4.19	0.85	−0.06*	−0.10**	−0.16**	0.60**	1				
6.PIM	4.49	0.64	−0.05	−0.07*	−0.09**	0.57**	0.60**	1			
7.LE	4.33	0.64	−0.05	−0.08**	−0.15**	0.82**	0.83**	0.89**	1		
8.MC	1.61	0.96	−0.08*	0.02	0.14**	−0.18**	−0.17**	−0.19**	−0.21**	1	
9.MPA	2.45	1.30	−0.04	0.14**	0.15**	−0.20**	−0.20**	−0.13**	−0.20**	0.51**	1

### 3.3 The mediating effect of maladaptive cognition

**Fig. 2:**
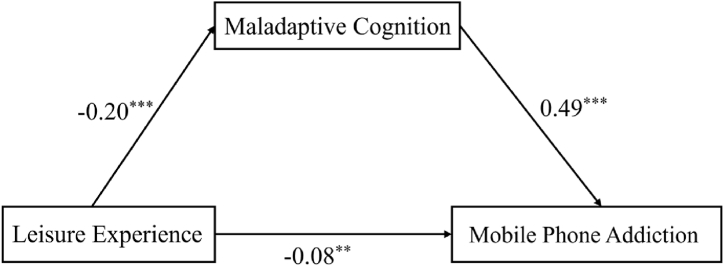
The Integrated Model. Path values are the path coefficients, **p < 0.01, ***p < 0.001.

To explore how leisure experience affects mobile phone addiction, we examined the mediation model proposed including leisure experience, maladaptive cognition and mobile phone addiction by using Model 4 of the SPSS Process component. The results of the regression analysis are displayed in Table 3 and Fig. 2. Specifically, the negative predictive effect of leisure experience on mobile phone addiction was significant (β = −0.18，p < 0.001). Hypothesis 1 was confirmed.

**Table 3:** Mediation of maladaptive cognition. *p < 0.05, **p < 0.01, ***p < 0.001.

Predictors	Equation 1 （Criterion: Maladaptive Cognition）			Equation 2 （Criterion: Mobile Phone Addiction）		
	β	t	95%CI	β	t	95%CI
Gender	−0.08	−2.73**	[-0.28, −0.05]	−0.01	−0.26	[-0.16, 0.12]
Age	−0.01	−0.45	[-0.07, 0.05]	0.12	4.51***	[0.09, 0.23]
FEC	0.11	3.54***	[0.08, 0.26]	0.06	2.01*	[0.00, 0.23]
LE	−0.20	−6.34***	[-0.39, −0.21]	−0.08	−2.86**	[-0.27, −0.05]
MC				0.49	17.38***	[0.58, 0.73]
R^2^	0.06			0.29		
F	16.70***			79.88***

After maladaptive cognition was included in the regression equation, leisure experience had a negatively residual direct effect on mobile phone addiction (β = −0.08, p < 0.01). The results demonstrated that leisure experience negatively predicted maladaptive cognition (β = −0.20, p < 0.001) and maladaptive cognition was positively associated with mobile phone addiction (β = 0.49, p < 0.001). Hypothesis 2 was confirmed.

## 4 Discussion

The decisive purpose of the study is to disclose how leisure experience influences mobile phone addiction and then, two hypotheses were tested. The results suggested that first, the prediction between leisure experience and mobile phone addiction is negative. Besides, maladaptive cognition may play a mediating role in both. Both of our hypotheses have been confirmed. In general, the study extends Davis's cognitive-behavioral model of PIU and contributes to empirical support for exploring the influence of leisure experience, maladaptive cognition and mobile phone addiction.

### 4.1 Leisure experience and mobile phone addiction

As expected, research findings demonstrated that leisure experience was correlated with mobile phone addiction and three dimensions to measure leisure experience were all negatively related to mobile phone addiction. That is to say, adolescents who perceived higher level of leisure experience were unlikely to overindulge in smartphones. Specifically, adolescents get more offline positive experiences when they have more independent choices, higher sense of accomplishment and stronger internal motivation in leisure. It also means that their psychological needs are satisfied and they don't need to ask for help in online world. On the contrary, the deficiency of the above experiences may induce frustration in satisfying needs in real world, which, according to the compensatory satisfaction theory, could trigger mobile phone addiction. In a previous study, high frequency mobile phone users were more extrinsically and less intrinsically motivated for leisure [40]. Once the comparative superiority of mobile phone and Internet is realized, adolescents build an online need satisfaction pattern, which makes them more prone to overuse mobile phones in order to compensate for the frustration of psychological needs satisfaction in real life [26]. Therefore, these positive experiences and evaluations act as important protective factors among adolescents [41].

The results conformed with the previous research, which suggests that adolescents may be less prone to be stuck with screen-based media when they obtain positive affective experience in leisure [42]. Apart from that, great leisure experiences could be effective in helping alleviate stress and depression, which come with some risks for problematic Internet use [31,43].

In the past, many studies have proved that leisure is beneficial to the reduction of mobile phone addiction. After analyzing 17 studies from China, India, Switzerland, Turkey and other different countries, researchers found that there was a moderate negative correlation between physical activity and mobile phone addiction in the young [44]. However, our study further confirmed that the positive psychological experience in leisure plays a special role in thwarting mobile phone addiction among adolescents.

On the whole, as a significant context for adolescents to learn several developmental skills, great leisure is often related to abundant positive effects, such as well-being, life satisfaction, social support and lower stress [[45], [46], [47]], which are associated with the reduction of problem behaviors [28,48,49].

Hence, our team is devoted to probing into the benefits of leisure on psychological health of adolescents, and how they arise.

### 4.2 The mediation of maladaptive cognition

Our research also discovered that the mediating role of maladaptive cognitive. To put it another way, the degree of maladaptive cognition and mobile phone addiction was lower in individuals with better leisure experience. Moreover, weaker maladaptive cognition predicted lower mobile phone addiction. There is no doubt that maladaptive cognition plays a key part in both the development and prevention of mobile phone addiction.

These results agreed with the cognitive-behavioral model mentioned above. From the model, distal contributory cause (leisure experience) may influence mobile phone addiction through proximal contributory cause (maladaptive cognition). Maladaptive cognition is derived from comparisons between the real world and the virtual world. It develops if an individual's perception of the virtual world is superior to that of the real world [31]. Based on previous studies, leisure is closely connected with social adaptation and self-efficacy, which are important parts of how people evaluate the world and themselves [50,51]. When adolescents have terrible psychological experiences in leisure, they have a negative view of the real world. It probably induces them to use the mobile phones to get more positive feedback from others. Such comparison will make adolescents form the maladaptive cognition that the Internet is better than reality, thus aggravating mobile phone dependence.

Furthermore, this model provides an insight into leisure experience and mobile phone addiction, which has been rarely discussed in numerous past studies, as far as we know. Past studies analyzing mediating variables between leisure and addiction has largely concentrated in distal factors such as subjective well-being, self-control, psychological capital while proximal factors were hardly discussed [41,52,53]. Compared with variables previously studied, maladaptive cognition, a proximal factor, is more closely connected with mobile phone addiction. This is also consistent with our study results. The inclusion of proximal factor in the study is essential to deepen our understanding of how leisure experience affects mobile phone addiction and therefore suggest targeted interventions.

## 5 Conclusions

In today's fast-paced world, more and more people find physical and psychological benefits of leisure. Especially during the COVID-19, many restrictions on interpersonal relationships have had adverse effects, such as being in a bad mood, using mobile phones too much [3,54,55].Running regularly, watching sports events, painting things that they are interested in at home have become effective ways for people to recharge their batteries. The public's concern for leisure has pushed forward the scientific process of it. How to maximize the positive effects of leisure in limited leisure time has become the interest of researchers. Therefore, they explored the relationship between leisure obstacles, leisure attitude, leisure motivation, leisure experience and some positive results.

We found that leisure experience was correlated with mobile phone addiction. Practically, the findings give some enlightenment for development and prevention of mobile phone addiction in adolescents. Parents and teachers should encourage students to participate more in leisure activities with certain skills, challenges, clear goals and feedback, such as sports and team games. Those enables adolescents to enhance their leisure experience, make their psychological needs fully satisfied in real life, and reduce excessive mobile phone use. In addition, considering the mediating role of maladaptive cognitive between leisure experience and mobile phone addiction, it is necessary to guide adolescents to perceive their irrational beliefs in leisure and rethink offline leisure and real life from more positive views. For example, Bob's team lost a basketball game today. He felt very sad, but he knew that he was not completely worthless in this game: I fully demonstrated my abilities, resolutely implemented the team's tactics, and my players have always encouraged each other. This was a very rewarding game, which was worth summarizing.

Theoretically, the influence of leisure experience on adolescents' mobile phone addiction was little explored. Based on prior research, it is momentous to underline the mediating function served by maladaptive cognition. The compensatory satisfaction theory and the cognitive-behavioral model of PlU are all supported in accordance with the study's results, which give empirical indication to the previous studies.

In our view, the wide range of advantages that leisure may generate deserves consideration, especially among adolescents.

## 6 Limitations

At the same time, there are some limitations in the study. We conducted the cross-sectional study, therefore, it is not suitable to judge the causal relationship between variables based on the research results. It could be further verified by longitudinal studies in the future. Second, adolescents' self-reported mobile phone addiction may be concealed. It is necessary to add other methods to make the results more objective, for example, observations from teachers may be appropriate. Besides, this study only collected data based on questionnaire survey, which could not deeply understand the change of adolescents' leisure experience in leisure participation. It is further investigated through interviews.

## Funding

This work was supported by the National Social Science Fund of China (Nos. 19BKS087).

## Ethics statement

This study strictly followed the Helsinki Declaration guidelines and was approved by the department of Psychology, Qingdao University (2,022,035). We guaranteed the informed consent of the subjects. Moreover, we protected subjects' privacy through data anonymity.

## Data availability statement

Considering the privacy of the participants, the data can only be provided according to reasonable requirements.

## CRediT authorship contribution statement

**Ximei Xia:** Supervision, Resources, Investigation, Funding acquisition, Conceptualization. **Shuhui Qin:** Writing – review & editing, Writing – original draft, Visualization, Validation, Project administration, Methodology, Investigation, Formal analysis, Data curation, Conceptualization. **Shiyin Zhang:** Methodology, Investigation, Conceptualization.

## Declaration of competing interest

The authors declare that they have no known competing financial interests or personal relationships that could have appeared to influence the work reported in this paper.
